# Relative Value of Different Medicines Ordinarily Employed in Syphilis

**Published:** 1847-09

**Authors:** 


*Relative Value of Different Medicines Ordinarily Empolyed in Syphilis.*—A long memoir on this subject by MM. Boys de Loury and Costilhes closes with the following propositions:

1. The preparations of gold and silver are worthless in constitutional syphilis.

2. The iodide of potassium has no influence upon the progress and duration of chancre, particularly the indurated variety: but in such cases mercury is to be preferred. The same may be said as respects buboes, mucous tubercles, papular and pustular syphilides.

On the other hand, iodide of potassium is invaluable in syphilitic disease of bones, and in syphilitic tubercles of the skin with or without ulceration. It may be stated in general terms that the iodide is successful in proportion to the anterior duration of the disease, and the degeneration of the constitutional powers.

Iodide of potassium acts like a charm in nocturnal pains. United with the bin-iodide of mercury, it is valuable in syphilitic cachexia, and in cases which have resisted ordinary mercurial treatment.

3. Mercurial fumigations are only available in healing local symptoms, and should not be employed as a means of affecting the system.

4. Mercurial frictions should be used only in those cases in which irritability of the mucous membranes forbids the internal administration of mercury.

5. The proto-iodide of mercury is the best medicine which can be employed as an antisyphilitic, as well in the primary as the consecutive symptoms of the disease.—*Ibid, from Gazette Mèdicale.*

